# Integrative Evaluation of Salt Tolerance in Cherry Rootstocks Using Phenotypic and Biochemical Markers

**DOI:** 10.3390/plants15050737

**Published:** 2026-02-28

**Authors:** Juan Zhang, Guanhua Lan, Feng An, Zhenfei Xing, Chenxue Lin, Yuliang Cai

**Affiliations:** 1 College of Horticulture, Northwest A&F University, Yangling 712100, China; 120050115@taru.edu.cn (J.Z.); xiaodai_an@nwafu.edu.cn (F.A.); 2 College of Horticulture and Forestry, Tarim University, Alar 843300, China; 10757241046@stumail.taru.edu.cn (G.L.); 10757241048@stumail.taru.edu.cn (Z.X.); 18099798491@163.com (C.L.); 3 National and Local Joint Engineering Laboratory of High-Efficiency and Good Quality Cultivation and Deep-Processing Technology of Characteristic Fruit Trees in Southern Xinjiang, Alar 843300, China; 4 Xinjiang Production & Construction Corps Key Laboratory of Facility Agriculture, Tarim University, Alar 843300, China

**Keywords:** *Prunus avium* L., phenotype, physiological responses, rootstock selection, salt stress tolerance

## Abstract

The sweet cherry has become a commercially significant fruit crop, yet its cultivation in Xinjiang is severely constrained by saline-alkali soils. To address this, selecting salt-tolerant rootstocks is vital for sustainable crop production in salinized soils. This study investigated the physiological and biochemical responses of five major rootstock cultivars (‘Mahaleb CDR-1’, ‘Gisela 6’, ‘Colt’, ‘Daqingye’, and ‘Krymsk5’) to a gradient of NaCl stress (0, 50, 100, 150, 200, 250, 300 mmol·L^−1^) under controlled environmental conditions. Key osmoprotectants and antioxidant systems showed a consistent trend across genotypes: the contents of soluble sugars, proteins, and proline, along with the activities of catalase (CAT), superoxide dismutase (SOD), and peroxidase (POD), initially increased under moderate stress but subsequently declined as salinity stress intensified. Specifically, CAT activity peaked at 150 mmol·L^−1^ NaCl in most genotypes, with significant increases ranging from 33.9% (‘Gisela 6’ at 100 mmol·L^−1^) to 45.52% (‘Colt’ at 150 mmol·L^−1^) compared to controls. SOD activity also reached maxima at 150 mmol·L^−1^ in most cultivars, increasing by 11.30% to 19.38% relative to controls, while POD activity exhibited peak values at 150–200 mmol·L^−1^, with increases of 4.12% to 10.45%. Notably, proline (PRO) accumulation peaked at 150 mmol·L^−1^ NaCl, with ‘Mahaleb CDR-1’ demonstrating the highest concentration (29.81 μg·g^−1^) and ‘Colt’ the lowest (25.85 μg·g^−1^). Conversely, the malondialdehyde (MDA) content, an indicator of membrane lipid peroxidation, increased progressively with increasing salinity. The cultivar ‘Colt’ exhibited the most severe membrane damage. Its MDA content under 300 mmol·L^−1^ NaCl stress was 80.84% higher than that under the control condition (0 mmol·L^−1^ NaCl). These results demonstrate that under moderate salt stress, the rootstocks activated adaptive responses, as evidenced by elevated osmoprotectant levels and enzymatic activity, which were ultimately suppressed under severe conditions. A comprehensive analysis of all physiological and biochemical indices allowed for a clear ranking of salt tolerance: ‘Mahaleb CDR-1’ > ‘Daqingye’ > ‘Krymsk5’ > ‘Gisela 6’ > ‘Colt’. This study provides a robust physiological basis for selecting salt-tolerant rootstocks in saline-alkaline regions and offers valuable insights for breeding programs aimed at enhancing stress resilience.

## 1. Introduction

Soil salinity is one of the major abiotic stressors that severely limits plant growth and productivity, particularly in arid and semi-arid regions [1,2,3]. As a global ecological challenge, soil salinization poses a significant threat to both environmental stability and the sustainability of agricultural systems. It is estimated that over 800 million hectares of land across more than 100 countries are affected by salinization, with the affected area continuing to expand [4]. Projections suggest that at least 20% of the world’s cultivated land will experience salinity-related degradation in the near future [5]. China ranks third globally in the extent of saline-alkaline soils, with soils accounting for approximately 25% of the nation’s total arable land. Within China, Xinjiang is particularly impacted due to its arid climate and limited precipitation. Saline-alkaline soils in Xinjiang constitute nearly one-third of the country’s total saline-alkaline area, with over 1.5 million hectares of arable land affected. Notably, more than 30% of this land is classified as severely salinized [6]. The salt composition in Xinjiang’s soils is complex, predominantly of the mixed sulphate-chloride type, which represents over 70% of the total salt content. Southern Xinjiang, particularly the Tarim Basin, exhibits the highest degree of salinization, comprising more than 60% of the region’s total saline-alkaline land.

NaCl (common salt) is one of the most prevalent salts contributing to soil salinization. Under NaCl-induced stress, the external water potential surrounding plant roots decreases, leading to osmotic stress. This osmotic imbalance is often followed by ionic stress due to the excessive accumulation of Na^+^ and Cl^−^ ions in plant tissues. The capacity for salt ion accumulation varies significantly among plant species; however, elevated concentrations of Na^+^ and Cl^−^ can exert ion-specific toxicity. This disrupts the plant’s internal water balance, lowers soil osmotic potential, and raises the relative osmotic potential of root cells, ultimately resulting in water deficit conditions. As a consequence, water uptake by the roots is hindered. Moreover, the influx of Na^+^ and Cl^−^ alters the electrochemical gradient across cellular membranes, leading to structural and compositional changes in the plasma membrane. Increased membrane permeability facilitates the leakage of essential intracellular solutes, including potassium (K^+^), phosphorus (P), and various organic compounds, thereby causing nutritional imbalances. Water deficit within plant tissues further impairs key physiological processes such as cell division, elongation, and differentiation. These disruptions manifest as reduced leaf expansion, diminished photosynthetic light absorption, and ultimately, a decline in plant dry biomass and growth [7,8,9]. Overall, the reduction in water and osmotic potential in leaves is closely linked to both the osmotic pressure and the concentration of salts in the rhizosphere [10].

Sweet cherry (*Prunus avium* L.), native to the coastal regions of the Black Sea and Caspian Sea, is widely recognized for its early flowering period—earning it the titles “the first branch of spring fruits” and “jewel fruit”. As one of the most important deciduous fruit tree species cultivated globally, sweet cherry holds substantial commercial value. In China, sweet cherries, as an early spring fruit, demonstrate strong growth potential. Consequently, optimizing suitable planting area and expanding cultivation zones are critical for promoting the healthy, rapid, and sustainable growth of China’s sweet cherry industry and supporting rural revitalization initiatives. Despite its commercial promise, sweet cherry is highly sensitive to soil conditions. It thrives best in soils with a pH range of 6.0–7.5 and exhibits limited tolerance to salinity. Grafting onto salt-tolerant rootstocks has proven to be an effective strategy for enhancing the species’ adaptability to saline environments. Therefore, the selection and breeding of high-quality, salt-tolerant rootstocks are of great importance for improving the salt resistance of sweet cherry trees, ultimately contributing to enhanced fruit quality and the long-term viability of cherry cultivation in challenging environments.

Previous studies on the salt tolerance of cherry rootstocks have primarily focused on several physiological and molecular mechanisms, including osmoregulatory and antioxidant enzyme systems [11,12], photosynthetic performance [13], ion homeostasis [14,15], and gene-level responses [16,17,18,19]. In the present study, five cherry rootstocks commonly used in Xinjiang—‘Daqingye,’ ‘Colt,’ ‘Mahaleb CDR-1,’ ‘Kremsk 5,’ and ‘Gisela 6’—were selected as model plants. By evaluating morphological traits, osmoregulatory compounds, antioxidant enzyme activity, and photosynthetic parameters under NaCl stress to reveal the effects of NaCl-induced salt stress, this study aims to elucidate the physiological and biochemical responses of different cherry rootstocks. The findings are expected to provide a scientific basis for selecting salt-tolerant rootstocks and supporting the sustainable cultivation of sweet cherry in saline-affected regions.

## 2. Results

### 2.1. Effect of NaCl Stress on the Growth and Phenotype of Different Cherry Rootstocks

**Figure 1:**
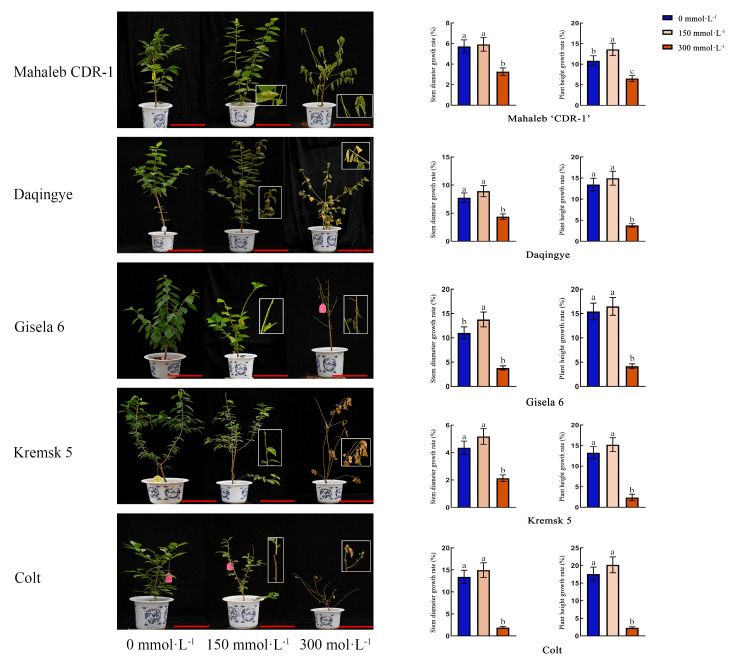
Effects of NaCl stress on the growth and morphology of five cherry rootstock genotypes. Plants of ‘Daqingye,’ ‘Colt,’ ‘Mahaleb CDR-1,’ ‘Kremsk 5’, and ‘Gisela 6’ were irrigated with 0, 150, or 300 mmol/L NaCl solutions for 28 days. Growth rates of plant height and stem diameter are shown. Error bars indicate the standard deviation of treatment means. Different lowercase letters (a, b, c) indicate significant differences at the 0.05 level.Scale bar: 15 cm.

Following 28 days of treatment with different concentrations of NaCl stress, the five cherry rootstocks exhibited phenotypic differences in salt stress symptoms (Figure 1). All rootstocks showed varying degrees of leaf chlorosis and growth inhibition under salt stress, but the severity differed significantly. As shown in the figure, under 150 mmol/L NaCl stress, all five cherry rootstocks displayed leaf yellowing and shedding. ‘Daqingye’ exhibited leaf curling and wilting, while ‘Gisela 6,’ ‘Krymsk5,’ and ‘Colt’ experienced more significant leaf drop. Under 300 mmol/L NaCl stress, ‘Mahaleb CDR-1’ and ‘Daqingye’ showed leaf withering and abscission. ‘Gisela 6,’ ‘Krymsk5,’ and ‘Colt’ demonstrated more severe symptoms, including widespread leaf margin scorching, severe defoliation, leaf necrosis, and even plant death, indicating pronounced salt sensitivity. Based on visual leaf symptoms and salt injury index scores (Table 1), the five rootstocks were preliminarily classified as follows: highly salt-tolerant ‘Mahaleb CDR-1’; salt-tolerant ‘Daqingye’; moderately salt-tolerant ‘Gisela 6’ and ‘Krymsk5’; and salt-sensitive ‘Colt’.

As shown in Figure 1, the growth rates of stem diameter and plant height in all five cherry rootstocks exhibited an initial increase followed by a decline as NaCl concentration increased. Under 150 mmol·L^−1^ NaCl treatment, the growth rates of both stem diameter and plant height in the five cherry rootstocks were higher than those of the control (0 mmol·L^−1^ NaCl). Specifically, the increases in stem diameter and plant height growth rates were as follows: ‘Daqingye,’ 1.17% and 1.49%; ‘Colt,’ 1.52% and 2.61%; ‘Krymsk5,’ 0.82% and 1.93%; ‘Gisela 6,’ 2.75% and 1.04%; and ‘Mahaleb CDR-1,’ 0.2% and 2.75% (*p* < 0.05). Under 300 mmol·L^−1^ NaCl treatment, the growth rates of stem diameter and plant height in all five cherry rootstocks were significantly lower than those of the control. The reductions were as follows: ‘Daqingye’ 3.36% and 9.71%; ‘Colt’ 11.52% and 15.26%; ‘Krymsk5’ 2.22% and 10.89%; ‘Gisela 6’ 7.22% and 11.25%; and ‘Mahaleb CDR-1’ 2.46% and 4.32% (*p* < 0.05). Compared with ‘Colt,’ ‘Mahaleb CDR-1’ exhibited a smaller reduction in plant height and stem diameter growth rates under 300 mmol·L^−1^ NaCl, demonstrating stronger stress tolerance. In contrast, ‘Colt’ was more sensitive to changes in salt concentration and showed the largest reductions in both plant height and stem diameter growth rates under 300 mmol·L^−1^ NaCl.

### 2.2. Effect of NaCl Stress on Root-to-Shoot Ratio (Fresh Weight) of Different Cherry Rootstocks

As shown in Table 2, the root-to-shoot (root-crown) ratios of the five cherry rootstocks initially increased and then decreased with rising NaCl concentrations; however, the magnitude and pattern of change varied among cultivars. ‘Mahaleb CDR-1,’ ‘Daqingye,’ and ‘Krymsk5’ achieved their maximum root-to-shoot ratios at 150 mmol·L^−1^ NaCl, with values of 2.11, 0.81, and 1.40, respectively. In contrast, ‘Gisela 6’ and ‘Colt’ reached their highest ratios at 100 mmol·L^−1^, with values of 1.51 and 1.28, respectively. At 300 mmol·L^−1^ NaCl, the root-to-shoot ratios of ‘Daqingye,’ ‘Gisela 6,’ and ‘Colt’ were not significantly different from each other. Among all rootstock cultivars, ‘Mahaleb CDR-1’ exhibited the highest root-to-shoot ratio at this concentration (1.85), whereas ‘Krymsk5’ had the lowest (0.51).

After 28 days of coercion, compared with the control, ‘Mahaleb CDR-1’ and ‘Daqingye’ showed increases of 25.57% and 10.53% in root-to-shoot ratio at 300 mmol·L^−1^ NaCl, respectively. In contrast, ‘Krymsk5,’ ‘Gisela 6,’ and ‘Colt’ showed substantial reductions of 28.58%, 73.85%, and 60.44%, respectively.

### 2.3. Effects of NaCl Stress on Photosynthetic Characteristics of Different Cherry Rootstocks

NaCl stress significantly affected the photosynthetic gas exchange parameters of the five cherry rootstocks. As NaCl concentration increased, the net photosynthetic rate (Pn), transpiration rate (Tr), and stomatal conductance (Gs) of all rootstocks showed a significant declining trend (Supplementary Tables S1–S3). After 28 days of treatment at 300 mmol·L^−1^ NaCl, ‘Mahaleb CDR-1’ exhibited the smallest reductions in Pn, Tr, and Gs (55.77%, 19.82%, and 58.31%, respectively) compared to their controls, indicating higher photosynthetic stability and superior water regulation (*p* < 0.05). In contrast, ‘Krymsk5’ showed the most pronounced decline in Pn (78.61%) (*p* < 0.05). At low salt concentrations (50–150 mmol·L^−1^), Gs changes in some rootstocks were not significant, whereas at high concentrations (200–300 mmol·L^−1^), no significant differences in Gs were observed among the rootstocks.

The response pattern of intercellular CO_2_ concentration (Ci) to NaCl stress differed from other parameters, showing an initial decrease followed by an increase as salt levels rose (Supplementary Tables S4). The turning point concentration varied among rootstocks: 100 mmol·L^−1^ for ‘Daqingye’, ‘Krymsk5’, and ‘Colt’; 150 mmol·L^−1^ for ‘Gisela 6’; and 200 mmol·L^−1^ for ‘Mahaleb CDR-1’. After 28 days at 300 mmol·L^−1^, Ci values in all rootstocks were higher than their respective controls, with ‘Mahaleb CDR-1’ showing the smallest increase (15.94%) and ‘Colt’ the largest (71.07%) (*p* < 0.05). This reflects differing degrees of stomatal limitation and photosynthetic response under high salinity among the varieties.

Overall, the analysis of photosynthetic parameters indicates that different cherry rootstocks exhibit varying levels of adaptability to salt stress. Among them, ‘Mahaleb CDR-1’ demonstrated relatively greater stability across all measured photosynthetic indices under saline conditions, suggesting a stronger physiological tolerance to salt stress.

### 2.4. Effects of NaCl Stress on Osmoregulatory Substances in the Leaves of Different Cherry Rootstocks

#### 2.4.1. Effect of NaCl Stress on Soluble Sugar Content in the Leaves of Different Cherry Rootstocks

**Figure 2:**
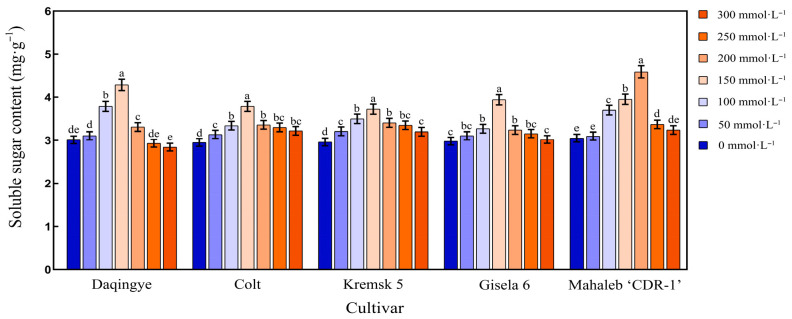
Effect of NaCl stress on soluble sugar content in the leaves of different cherry rootstocks [‘Daqingye,’ ‘Colt,’ ‘Mahaleb CDR-1,’ ‘Kremsk 5,’ and ‘Gisela 6’]. Error bars indicate the SD of treatment means. Different letters on the bars indicate significant differences among the treatments (*p* < 0.05).

As shown in Figure 2, the soluble sugar content in the leaves of different cherry rootstocks initially increased and then declined with increasing NaCl concentration. Under 150 mmol·L^−1^ NaCl stress, ‘Daqingye,’ ‘Gisela 6,’ ‘Krymsk5,’ and ‘Colt’ reached their peak soluble sugar contents of 4.29%, 3.94%, 3.72%, and 3.79%, respectively (*p* < 0.05). Notably, ‘Mahaleb CDR-1’ exhibited a delayed peak (4.59%) at 200 mmol·L^−1^, indicating a distinct salt-tolerant response. After 28 d of NaCl stress at 300 mmol·L^−1^, ‘Daqingye’ showed a 5.64% reduction in soluble sugar content compared to the control, whereas ‘Colt,’ ‘Krymsk5,’ ‘Gisela 6,’ and ‘Mahaleb CDR-1’ exhibited increases of 2.30%, 2.28%, 2.03%, and 2.30%, respectively (*p* < 0.05). Under 150 mmol·L^−1^ NaCl stress, the changes in physiological parameters of ‘Colt’ and ‘Kremsk 5’ were more gradual compared to those of ‘Daqingye’, ‘Mahaleb CDR-1’, and ‘Gisela 6’ (Supplementary Figure S1).

#### 2.4.2. Effect of NaCl Stress on Soluble Protein Content in the Leaves of Different Cherry Rootstocks

**Figure 3:**
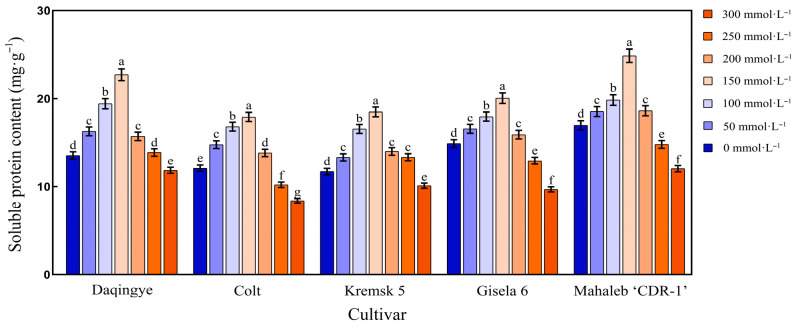
Effect of NaCl stress on soluble protein content in the leaves of different cherry rootstocks [‘Daqingye,’ ‘Colt,’ ‘Mahaleb CDR-1,’ ‘Kremsk 5,’ and ‘Gisela 6’]. Error bars indicate the SD of treatment means. Different letters on the bars indicate significant differences among the treatments (*p* < 0.05).

As shown in Figure 3, the soluble protein content in the leaves of different cherry rootstocks exhibited a trend of initial increase followed by a decline under increasing NaCl concentrations. At 150 mmol·L^−1^ NaCl, ‘Daqingye,’ ‘Gisela 6,’ ‘Krymsk5,’ ‘Colt,’ and ‘Mahaleb’ reached peak soluble protein contents of 22.69%, 20.05%, 18.5%, 17.91%, and 24.86%, respectively (*p* < 0.05). At 300 mmol·L^−1^ NaCl, the soluble protein contents of ‘Daqingye,’ ‘Gisela 6,’ ‘Krymsk5,’ ‘Colt,’ and ‘Mahaleb CDR-1’ decreased by 12.69%, 34.95%, 13.68%, 30.49%, and 29.04%, respectively, relative to those in the control (*p* < 0.05). Notably, at 300 mmol·L^−1^, ‘Mahaleb CDR-1’ maintained the highest soluble protein content, being 1.02-, 1.24-, 1.19-, and 1.43-fold higher than those of ‘Daqingye,’ ‘Gisela 6,’ ‘Krymsk5,’ and ‘Colt,’ respectively (*p* < 0.05). Under treatment with 150 mmol·L^−1^ NaCl, the growth rates of ‘Daqingye’ and ‘Mahaleb CDR-1’ were significantly higher than those of the other tested cultivars (Supplementary Figure S2).

#### 2.4.3. Effect of NaCl Stress on PRO Content in the Leaves of Different Cherry Rootstocks

**Figure 4:**
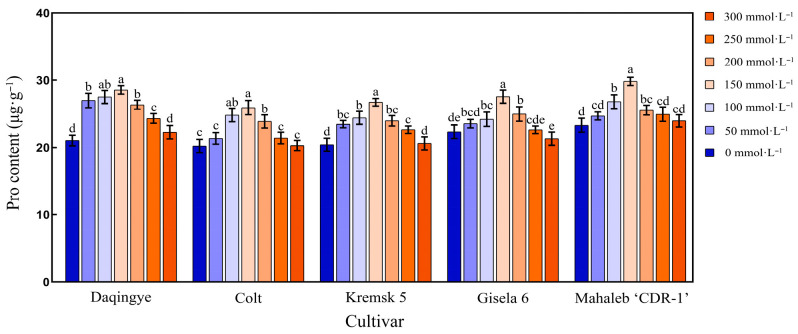
Effect of NaCl stress on Pro content in the leaves of different cherry rootstocks [‘Daqingye,’ ‘Colt,’ ‘Mahaleb CDR-1,’ ‘Kremsk 5,’ and ‘Gisela 6’]. Error bars indicate the SD of treatment means. Different letters on the bars indicate significant differences among the treatments (*p* < 0.05).

PRO accumulation in the leaves of the five cherry rootstock cultivars exhibited a trend of initial increase followed by a decline as NaCl concentrations increased; the magnitude of change varied significantly among cultivars (Supplementary Figure S3). As shown in Figure 4, all cultivars reached their peak PRO levels at 150 mmol·L^−1^ NaCl, with ‘Mahaleb CDR-1’ displaying the highest content (29.81 μg·g^−1^) and ‘Colt’ the lowest (25.85 μg·g^−1^) (*p* < 0.05). When NaCl concentrations exceeded 150 mmol·L^−1^, PRO content in all cultivars declined. After 28 d of NaCl treatment at 300 mmol·L^−1^, the content in ‘Daqingye,’ ‘Colt,’ ‘Krymsk5,’ and ‘Mahaleb CDR-1’ increased by 6.03%, 0.61%, 0.96%, and 3.23%, respectively, compared to that in the control, whereas ‘Gisela 6’ exhibited a 4.62% decrease relative to the control (*p* < 0.05).

#### 2.4.4. Effect of NaCl Stress on MDA Content in the Leaves of Different Cherry Rootstocks

**Figure 5:**
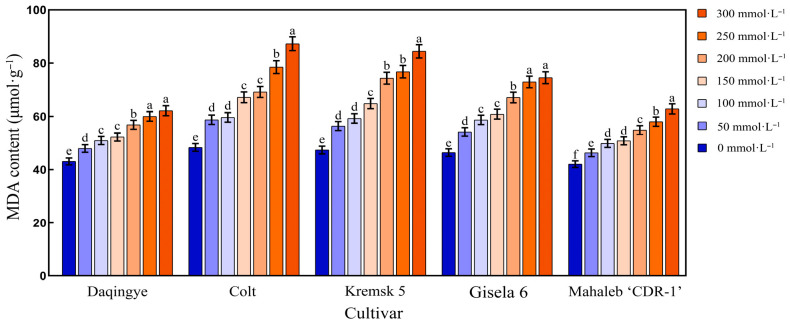
Effect of NaCl stress on MDA content in the leaves of different cherry rootstocks [‘Daqingye,’ ‘Colt,’ ‘Mahaleb CDR-1,’ ‘Kremsk 5’, and ‘Gisela 6’]. Error bars indicate the SD of treatment means. Different letters on the bars indicate significant differences among the treatments (*p* < 0.05).

As shown in Figure 5, after 28 d of NaCl stress, the MDA content in the leaves of different cherry rootstocks exhibited a continuous increasing trend with increasing NaCl concentrations. This accumulation pattern suggests that membrane lipid peroxidation intensified under salt stress, reflecting enhanced oxidative damage. The magnitude of MDA accumulation varied across cultivars (Supplementary Figure S4). Notably, ‘Colt’ exhibited a pronounced increase in MDA content, while ‘Daqingye’ and ‘Mahaleb CDR-1’ showed relatively moderate and stable changes across treatments. At 300 mmol·L^−1^ NaCl, ‘Mahaleb CDR-1’ and ‘Daqingye’ showed increases of 44.10% and 49.33%, respectively, compared to their controls. In contrast, ‘Gisela 6,’ ‘Krymsk5,’ and ‘Colt’ showed significantly higher MDA levels, with increases of 60.82%, 78.46%, and 80.84%, respectively (*p* < 0.05).

### 2.5. Effect of NaCl Stress on Antioxidant Enzyme Systems in the Leaves of Different Cherry Rootstocks

#### 2.5.1. Effect of NaCl Stress on CAT Activity in the Leaves of Different Cherry Rootstocks

**Figure 6:**
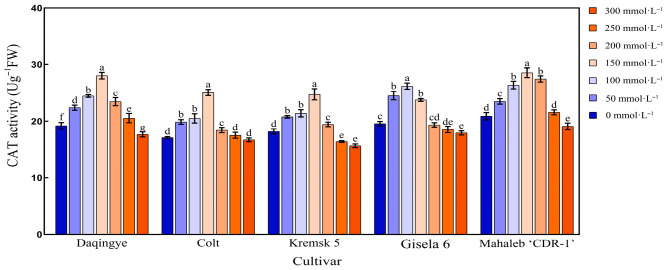
Effect of NaCl stress on catalase (CAT) activity in the leaves of different cherry rootstocks [‘Daqingye,’ ‘Colt,’ ‘Mahaleb CDR-1,’ ‘Kremsk 5’, and ‘Gisela 6’]. Error bars indicate the SD of treatment means. Different letters on the bars indicate significant differences among the treatments (*p* < 0.05).

As shown in Figure 6, CAT activity in the leaves of all five cherry rootstocks exhibited a unimodal response to increasing NaCl concentrations—initially rising and then declining. However, the concentration at which peak CAT activity occurred varied among genotypes (Supplementary Figure S5). ‘Gisela 6’ reached its maximum CAT activity at 100 mmol·L^−1^ NaCl, displaying a significant increase of 33.9% compared to the control. In contrast, ‘Mahaleb CDR-1,’ ‘Daqingye,’ ‘Krymsk5,’ and ‘Colt’ exhibited peak CAT activities at 150 mmol·L^−1^, with increases of 37.67%, 45.13%, 36.55%, and 45.52%, respectively, relative to their controls. After 28 d of NaCl stress at 300 mmol·L^−1^, ‘Mahaleb CDR-1’ maintained the highest CAT activity (18.5 U·g^−1^), exceeding that of ‘Daqingye,’ ‘Colt,’ ‘Krymsk5,’ and ‘Gisela 6’ by 1.07-, 1.13-, 1.22-, and 1.06-fold, respectively (*p* < 0.05).

#### 2.5.2. Effect of NaCl Stress on SOD Activity in the Leaves of Different Cherry Rootstocks

**Figure 7:**
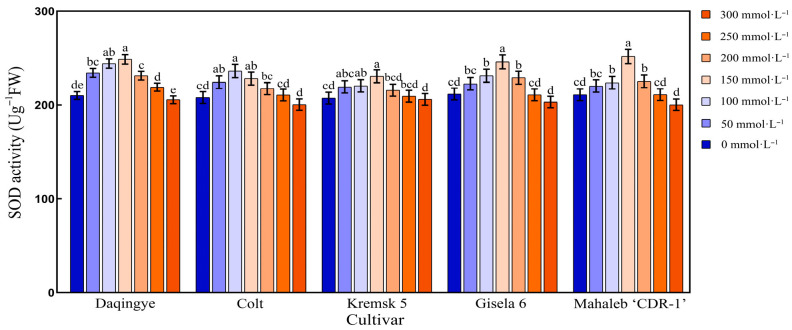
Effect of NaCl stress on SOD activity in the leaves of different cherry rootstocks [‘Daqingye,’ ‘Colt,’ ‘Mahaleb CDR-1,’ ‘Kremsk 5’, and ‘Gisela 6’]. Error bars indicate the SD of treatment means. Different letters on the bars indicate significant differences among the treatments (*p* < 0.05).

As shown in Figure 7, SOD activity in the leaves of all five cherry rootstocks followed a unimodal pattern in response to increasing NaCl concentrations, characterized by an initial increase and subsequent decline. Among the cultivars, ‘Colt’ reached its peak SOD activity (236.37 U·g^−1^) at 100 mmol·L^−1^ NaCl, representing a 13.66% increase relative to the control. In contrast, ‘Mahaleb CDR-1,’ ‘Daqingye,’ ‘Krymsk5,’ and ‘Gisela 6’ exhibited maximum SOD activity at 150 mmol·L^−1^, with increases of 19.38%, 18.37%, 11.30%, and 16.22%, respectively, relative to their corresponding controls (*p* < 0.05). Compared with other cultivars, the SOD activity of ‘Colt’ reached its peak at a relatively lower salt concentration (100 mmol·L^−1^) (Supplementary Figure S6).

#### 2.5.3. Effect of NaCl Stress on POD Activity in the Leaves of Different Cherry Rootstocks

**Figure 8:**
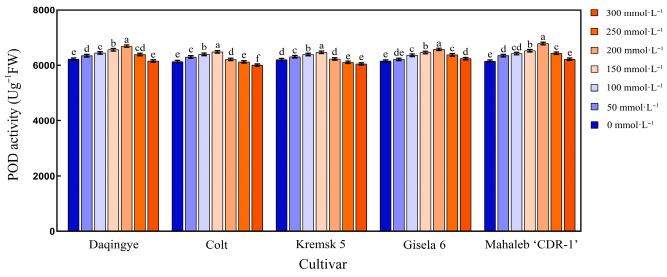
Effect of NaCl Stress on POD activity in the leaves of different cherry rootstocks [‘Daqingye,’ ‘Colt,’ ‘Mahaleb CDR-1,’ ‘Kremsk 5’, and ‘Gisela 6’]. Error bars indicate the SD of treatment means. Different letters on the bars indicate significant differences among the treatments (*p* < 0.05).

As shown in Figure 8, POD activity in the leaves of all five cherry rootstocks exhibited a characteristic “low-promotion and high-inhibition” unimodal response under increasing NaCl stress. ‘Krymsk5’ and ‘Colt’ reached their peak POD activity at 150 mmol·L^−1^ NaCl, showing significant increases of 4.12% and 5.60%, respectively, compared to the control. In contrast, ‘Mahaleb CDR-1,’ ‘Daqingye,’ and ‘Gisela 6’ demonstrated delayed peak responses at 200 mmol·L^−1^ NaCl, with POD activity increases of 10.45%, 7.56%, and 6.78%, respectively, relative to their corresponding controls (*p* < 0.05) (Supplementary Figure S7).

### 2.6. Evaluation of Salt Tolerance in Different Cherry Rootstocks

#### 2.6.1. Correlation Analysis

**Figure 9:**
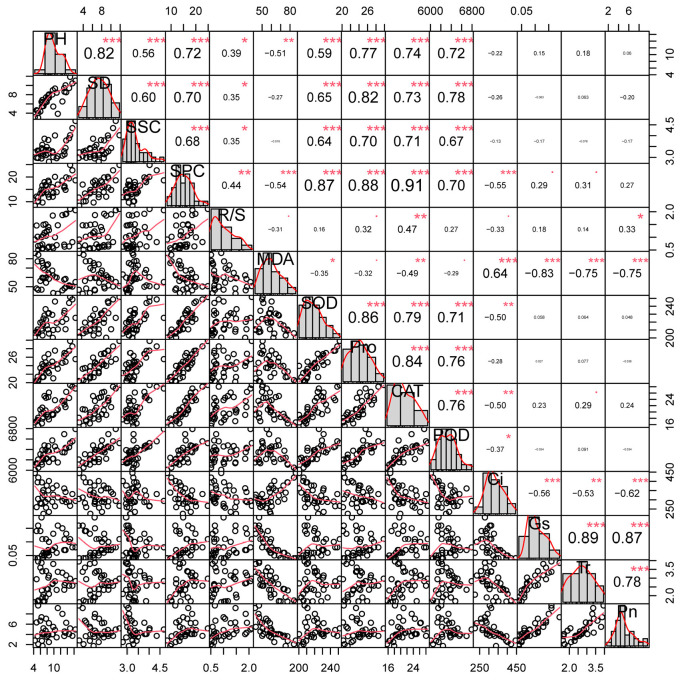
Correlation of Various Parameters in Cherry Rootstocks Under NaCl Stress. Note:·means significantly correlation, *p* < 0.1; * means significantly correlation, *p* < 0.05; ** means extremely correlation, *p* < 0.01; *** means extremely correlation, *p* < 0.001. Abbreviations: PH, plant height; SD, stem diameter; SSC, soluble sugar content; SPC, soluble protein content; R/S, root-to-shoot ratio.

As shown in Figure 9, correlation analysis revealed that key parameters formed distinct, interrelated groups. Growth parameters (plant height and stem diameter), osmotic adjustment substances (soluble sugars, soluble proteins, and proline), and the antioxidant enzyme system (SOD, POD, and CAT) were all significantly positively correlated with each other. In contrast, the oxidative stress marker MDA was negatively correlated with these beneficial traits and with photosynthetic parameters (Gs, Tr, Pn). Notably, Ci showed a significant negative correlation with the antioxidant enzymes and photosynthesis. These results indicate that under salt stress, robust growth is associated with enhanced osmotic adjustment, a coordinated antioxidant response, and maintained photosynthetic activity, while oxidative damage (MDA) is linked to the deterioration of these functions.

#### 2.6.2. Membership Function Analysis

To comprehensively assess the salt tolerance of different cherry rootstocks, a membership function analysis was performed using ten key physiological and biochemical indicators: plant height growth rate, stem diameter growth rate, root-to-shoot ratio, MDA content, Pro content, SOD activity, POD activity, CAT activity, soluble sugar content, and soluble protein content. For each rootstock, the average membership function values under NaCl stress were calculated across all indicators to generate a composite salt tolerance score.

As shown in Table 3, the comprehensive evaluation based on membership function values revealed the following salt tolerance ranking among the five cherry rootstocks (from strongest to weakest): ‘Mahaleb CDR-1’ > ‘Daqingye’ > ‘Krymsk5’ > ‘Gisela 6’ > ‘Colt’.

## 3. Discussion

### 3.1. Salt Stress and Plant Growth

Key morphological traits such as plant height, stem diameter, and root-to-shoot ratio are direct indicators of growth status and are widely used to assess seedling development. Under NaCl stress, salt accumulation disrupts root development and the uptake and transport of water and essential minerals, ultimately inhibiting shoot growth [20,21]. In this study, increasing NaCl stress concentrations resulted in a growth pattern characterized by an initial stimulation followed by decline in both plant height and stem diameter. At higher salt concentrations, growth was significantly suppressed. This trend is consistent with findings in olive trees subjected to salt stress, as reported by K. Chartzoulakis et al., and underscores the detrimental impact of excessive salinity on plant development [22].

### 3.2. Salt Stress and Biomass

Biomass allocation is one of the plastic adaptive mechanisms plants employ to cope with salt stress, generally conforming to the predictions of optimal allocation theory [23,24]. Roots serve as the primary organs for nutrient and water acquisition in plants, and their biomass directly determines the supply efficiency of resources to the aboveground parts. A higher root-to-shoot ratio generally indicates a well-developed root architecture, which not only improves the plant’s capacity for water and nutrient absorption but also enhances its physiological adaptability and stress tolerance under adverse environmental conditions [25,26,27]. In this study, the fresh weight root-to-shoot ratio of all cherry rootstock cultivars initially increased and subsequently declined with rising NaCl concentrations. Notably, the threshold concentration at which the ratio began to decrease varied among cultivars. ‘Colt’ and ‘Gisela 6’ exhibited peak root-to-shoot maximum ratios at 100 mmol·L^−1^ NaCl, whereas ‘Mahaleb CDR-1,’ ‘Daqingye,’ and ‘Krymsk5’ reached their maxima at 150 mmol·L^−1^ NaCl. These results suggest genotypic differences in biomass allocation strategies in response to salt stress.

### 3.3. Salt Stress and Plant Photosynthesis

Photosynthesis is a vital physiological process through which plants acquire energy, and it is highly sensitive to salt stress. Numerous studies have demonstrated that salinity leads to reductions in key photosynthetic parameters such as Pn, Gs, and Tr to varying extents [28,29]. The observed declines in photosynthesis performance under NaCl stress can be attributed to both stomatal and non-stomatal factors [30]. In the early stages of NaCl exposure, Na^+^ ions enter the plant system through the transpirational stream, leading to decreased transpiration and subsequent stomatal closure [31]. Prolonged stomatal closure limits CO_2_ uptake, and sustained declines in transpiration contribute to the accumulation of toxic ions, significantly impairing carbon assimilation efficiency. This study found that as the NaCl stress concentration increased, the net photosynthetic rate, transpiration rate, and stomatal conductance of cherry rootstocks all showed a decreasing trend. This is consistent with the photosynthetic performance of walnut rootstock, rapeseed and black locust seedlings under salt stress [32,33,34]. These reductions may be attributed to both direct and indirect effects of salt stress. Direct effects include damage to the photosynthetic apparatus, such as chloroplast degradation and reduced activity of essential enzymes (e.g., Rubisco and PEP carboxylase). Additionally, the accumulation of Na^+^ disrupts ion homeostasis within chloroplasts, further impairing photosynthetic function. Indirect effects involve salt-induced inhibition of plant growth and compromised root water uptake capacity. Elevated soil salinity lowers the water potential of the soil, triggering stomatal closure to minimize water loss, which further decreases stomatal conductance. Notably, Ci exhibited a biphasic pattern, initially declining and then increasing as NaCl stress intensified. This pattern likely reflects an initial limitation in CO_2_ diffusion caused by stomatal closure, despite unsynchronized declines in photosynthetic rate. As salinity stress persists, photosynthetic capacity deteriorates more rapidly than the restriction in CO_2_ influx, resulting in a subsequent rise in Ci.

### 3.4. Salt Stress and Osmotic Adjustment Substances

Osmotic adjustment in plants involves the accumulation of compounds such as carbohydrates, nitrogen-containing substances, and organic acids [35]. Studies by Navarro et al., Zheng et al., and Suarez Previous studies have shown that osmotic and elastic adjustments enable plants to maintain turgor pressure under conditions of low leaf water potential and reduced relative water content [36,37,38]. The extent of osmotic adjustment varies significantly across species and is influenced by factors such as the rate of stress imposition and plant developmental stage [39,40]. Under salinity-induced water stress or other abiotic stresses, plants respond by actively accumulating organic and inorganic solutes. This increases the solute concentration within cells, lowers cellular water potential, and helps maintain adequate hydration and turgor pressure—crucial for sustaining metabolic activity under stress. Organic osmolytes synthesized within the cell, such as PRO and soluble sugars, as well as inorganic ions absorbed from the external environment, both contribute to this osmotic adjustment process.

Under salt stress conditions, osmotic adjustment in plants is facilitated by the accumulation of substances such as soluble sugars, soluble proteins, and PRO, which help maintain cellular osmotic balance [41]. Soluble sugars not only function as readily available energy sources under stress conditions but also contribute significantly to membrane stabilization. Previous studies have reported a gradual increase in soluble sugar content under salt stress in various plant species, including Wheat Seedlings, Alfalfa, and Cucumis hystrix, which is consistent with the findings of the present study [42,43,44]. Herein, increasing NaCl concentrations led to a continuous rise in soluble sugar content in the leaves of all five cherry rootstocks. In contrast, soluble protein content displayed a unimodal response, initially increasing and then declining, with peak values occurring at different NaCl concentrations depending on the cultivar.

Under salt stress, the accumulation of PRO plays a vital role in regulating osmotic potential, thereby mitigating cellular damage caused by excessive water loss. In the present study, increasing NaCl concentrations led to a characteristic “low-promotion and high-inhibition” dose–response pattern of PRO accumulation across all cherry rootstock cultivars, with an initial increase followed by a decline. This trend may be attributed to metabolic collapse or suppression of PRO biosynthesis under prolonged or severe stress conditions. The observed decline in PRO content at high NaCl concentrations (>150 mmol·L^−1^) likely reflects impaired metabolic function and disrupted energy balance, potentially resulting from damage to membrane systems and subsequent physiological dysfunction.

As a key byproduct of membrane lipid peroxidation, MDA serves as widely recognized indicator of oxidative damage and plant resilience under salt stress, with its accumulation level generally inversely associated with stress tolerance [45,46]. In this study, MDA content in the leaves of all cherry rootstocks demonstrated a cumulative increase with rising NaCl concentrations, indicating that salt-induced lipid peroxidation intensified progressively under higher salinity levels. These results are consistent with previously reported MDA accumulation patterns under salt stress in Glycine max L., Cucumber, Potato, and Cherry rootstocks [47,48,49].

### 3.5. Salt Stress and Antioxidant Defense System

To prevent oxidative cellular damage, plants possess an intrinsic antioxidant enzyme system comprising SOD, POD, and CAT, which are ubiquitous in plant tissues. This system plays a central role in scavenging hydrogen peroxide (H_2_O_2_), a reactive oxygen species (ROS) produced during metabolic processes, thereby preventing its excessive accumulation and subsequent oxidative injury. Consequently, the activity levels of these antioxidant enzymes are closely associated with the plant’s tolerance to abiotic stress. To mitigate free radical-induced damage to vital biomacromolecules, plants typically enhance antioxidant enzyme activities, thereby improving their capacity to neutralize ROS. In this study, the activities of SOD, POD, and CAT in cherry rootstocks under NaCl stress exhibited a characteristic unimodal pattern—initially increasing and subsequently declining with rising salinity. This is consistent with previous findings on Medicago sativa, Astragalus membranaceus and Eucalyptus under salt stress [50,51].

### 3.6. Limitations of NaCl-Based Salt Tolerance Evaluation in Cherry Rootstocks and Future Perspectives

This study primarily evaluated the salt tolerance of cherry rootstocks based on their growth and physiological responses to NaCl stress. However, the actual salt composition in the Xinjiang southern region’s soil is complex, characterized mainly by sulfate-chloride mixed salinity with an accumulation of alkaline salts such as sodium bicarbonate. Since this experiment was conducted under controlled laboratory conditions using a single salt stressor, the findings may not accurately reflect the combined stress effects of multiple salts (e.g., Na_2_SO_4_, NaCl, NaHCO_3_) present in the natural saline-alkaline soils of southern Xinjiang, potentially leading to biased assessments of salt tolerance. Therefore, future research should focus on simulating mixed saline-alkaline stress conditions typical of the region—incorporating sulfates, chlorides, and bicarbonates—to systematically compare rootstocks’ physiological responses to neutral versus alkaline salt stress. Integrating transcriptomic and metabolomic analyses could further elucidate the gene regulatory networks underlying rootstock adaptation to combined saline-alkaline stress.

## 4. Materials and Methods

### 4.1. Plant Material

This experiment employed a completely randomized block design using five two-year-old cherry rootstock cultivars commonly utilized in sweet cherry production: ‘Daqingye’ and ‘Colt’ (vigorous rootstocks), ‘Mahaleb CDR-1’ (semi-dwarfing rootstock), and ‘Krymsk5’ and ‘Gisela 6’ (semi-vigorous rootstocks). The experiment was conducted over a two-year period (2024–2025) in a controlled multi-span greenhouse environment at the Horticultural Experimental Station of Tarim University (81.29° E, 40.54° N), located in Alar City, Xinjiang, China.

All plant materials were propagated through stem cuttings. Following successful rooting, the seedlings were initially transplanted into nutrient pots for preliminary cultivation. Once the seedlings attained a height of 15–20 cm and developed 3–5 healthy buds, uniformly growing, disease-free seedlings were selected for transplantation into experimental pots. These pots, each with a bottom-perforated design and a capacity of 5 L (30 × 30 cm), were filled with a growth substrate composed of peat and river sand mixed in a 1:1 volumetric ratio (total 5 kg, pH 7.0). Each pot contained a single plant. Salt stress treatments were administered by uniformly applying NaCl solutions at each concentration gradient to all walnut genotype seedlings every morning (8:00–10:00). Irrigation was conducted once per week, with 300 mL of solution applied per pot each time, for a total of four treatment cycles. To prevent leakage of the saline solution, appropriately sized saucers were placed under the pots, and any solution collected in the saucers was reapplied to the respective pot. Conventional seedling management practices were maintained throughout the experimental period.

### 4.2. Experimental Design

Following transplantation, all seedlings were irrigated with Hoagland nutrient solution to ensure uniform establishment. After a four-week acclimatization period, salt stress treatments were initiated using a single-salt NaCl solution. A randomized single-plant plot design was employed. Each treatment consisted of 15 replicate pots, with seven NaCl concentration gradients applied: 0 (control), 50, 100, 150, 200, 250, and 300 mmol·L^−1^. Irrigation was carried out once every seven days between 19:00 and 20:00, with each pot receiving 1500 mL of the respective salt solution. To minimize water loss and maintain consistent soil salinity, bottom trays were installed beneath each pot to collect leachate. The collected saline solution was periodically returned to the pots. Baseline measurements of plant height and stem diameter were taken prior to the initiation of salt treatments. These parameters were reassessed after 28 d of treatment to evaluate the morphological responses of different rootstocks to NaCl-induced changes.

### 4.3. Measurement Methods

#### 4.3.1. Growth Parameter Measurements

(1) Plant Height: The vertical distance from the rootstock base to the shoot apex of each seedling was measured using a standard measuring tape.
(2) Stem Diameter: Stem thickness was measured 1 cm above the root collar using a digital vernier caliper.
(3) Aboveground/Belowground Fresh and Dry Weights: Five seedlings per treatment group were sampled. Fresh weights of aboveground and belowground parts were recorded immediately using an electronic balance. For dry weight determination, samples were placed in kraft paper bags and subjected to initial heat treatment in oven at 105 °C for 15 min to deactivate enzymes, followed by drying at 80 °C for 24 h or until a constant weight was achieved. Final dry weights were then measured.
(4) Growth Rate of Plant Height and Stem Diameter (%): Growth rates were calculated using the following formula:

Growth Rate (%) = [(Plant height/stem diameter after salt treatment) − (Plant height/stem diameter before salt treatment)]/(Plant height/stem diameter before salt treatment) × 100%

(5) Root-to-Shoot Ratio: The ratio of belowground to aboveground biomass was calculated as:

Root-to-Shoot Ratio = (Belowground dry/fresh weight)/(Aboveground dry/fresh weight)

(6) Leaf Salt Injury Index (SI): On the 28th day of treatment, visible salt injury symptoms on leaves were assessed for plants in the 300 mmol·L^−1^ NaCl treatment group.

Assessment was conducted on all fully expanded leaves of each plant. The severity of injury was rated by at least two independent observers according to a five-grade scale adapted from Liu et al. [52]. The classification criteria were as follows:

Grade 0: No visible symptoms of salt injury.

Grade 1: Slight yellowing of leaf tips, margins, or veins.

Grade 2: Approximately half of the leaf tips and margins exhibit signs of scorching.

Grade 3: Severe scorching on most leaves accompanied by partial defoliation.

Grade 4: Extensive dieback, complete defoliation, and plant death.

Salt Injury Index (SI) = [(1 × S_1_ + 2 × S_2_ + 3 × S_3_ + 4 × S_4_)/(4 × Total number of plants)] × 100%, where S_1_–S_4_ represent the number of plants in each respective injury grade.

#### 4.3.2. Photosynthetic Parameter Measurements

Photosynthetic parameters were assessed using an LI-6400XT portable photosynthesis system (LI-COR Inc., Lincoln, NE, USA). Measurements were taken once prior to the initiation of salt treatment, as well as on days 7, 14, and 21 after salt application. The following parameters were recorded: Pn, Gs, Ci, Tr. All measurements were conducted under standardized environmental conditions: photosynthetically active radiation (PAR) at 2000 μmol·m^−2^·s^−1^, leaf chamber temperature at 32 °C, and CO_2_ concentration at 380 ± 10 μmol·mol^−1^. For each plant and at each time point, three fully expanded, healthy leaves (specifically, the second, fourth, and sixth leaves from the apex) were selected for measurement. Each treatment was replicated three times.

#### 4.3.3. Measurement of Antioxidant and Osmotic Adjustment-Related Physiological Parameters

The physiological indicators related to osmotic adjustment and antioxidative defense were quantified using the following methods:

Soluble Sugar Content: Determined using the anthrone colorimetric method [53]. Absorbance was measured at 630 nm, and soluble sugar concentration was calculated using a glucose standard curve.

Soluble Protein Content: Assessed via the Coomassie Brilliant Blue G-250 assay [53]. Absorbance was measured at 590 nm, and soluble protein content was determined using a (BSA) standard curve.

MDA Content: Measured using the thiobarbituric acid (TBA) assay [53]. Absorbance values at 520 nm were used to calculate MDA content employing a standard curve.

Pro Content: Quantified via the acidic ninhydrin assay [53]. Absorbance was measured at 515 nm, and PRO content was calculated using a standard curve.

The activities of superoxide dismutase (SOD), peroxidase (POD), and catalase (CAT) were determined using assay kits according to the manufacturer’s instructions (Comin Biotechnology, Suzhou, China) and following the method described by Yang et al. [54].

### 4.4. Statistical Analysis

All experimental data were statistically analyzed using Microsoft Excel 2020 and DPS v7.05 software. One-way analysis of variance (ANOVA) was performed to evaluate significant differences among treatments, and Duncan’s new multiple range test (α = 0.05) was applied for post hoc comparisons among treatments. Correlation analysis plots were generated using Origin 2022.

Comprehensive Evaluation of Salt Tolerance Using the Fuzzy Membership Function Method. The average membership function method (equal weight method) was used for evaluation, in which all measured indicators (such as plant height, biomass, SOD activity, proline content, etc.) were directly standardized (by calculating membership degree values) and then simply averaged to obtain a comprehensive evaluation score [55,56].

(1) To quantitatively assess the salt tolerance of the five cherry rootstocks under different NaCl concentrations, the fuzzy membership function method was employed. The membership function value for each physiological or morphological parameter was calculated using the following formula:

For positively correlated indicators (i.e., higher values indicate better salt tolerance):

X (f) = (X − X_min_)/(X_max_ − X_min_)

For negatively correlated indicators (i.e., higher values indicate poorer performance):

X (f) = 1 − (X − X_min_)/(X_max_ − X_min_)

where

X is the measured value of the indicator in a specific treatment,

X_max_ and X_min_ are the maximum and minimum observed values of the indicator across all treatments, respectively.

After calculating the membership function values for all indicators, they were summed and averaged to derive a comprehensive evaluation index for each rootstock. A higher index value reflects stronger salt tolerance.

## 5. Conclusions

In this study, the physiological and biochemical responses of different cherry rootstocks to salt stress varied significantly. The results of this study demonstrate significant differences in the physiological and biochemical responses of different cherry rootstocks to salt stress. Among them, ‘Mahaleb CDR-1’ and ‘Daqingye’ exhibited strong salt tolerance, maintaining higher levels of osmotic adjustment substances and lower accumulation of MDA under high salt stress. This indicates that they likely alleviated salt-induced membrane lipid peroxidation damage through the synergistic enhancement of osmotic regulation and antioxidant defense systems. This indicates that it effectively alleviated salt stress damage through the synergistic enhancement of osmotic adjustment and antioxidant defense. In contrast, sensitive varieties such as ‘Colt’ showed weaker osmotic regulation and antioxidant capacity, significant MDA accumulation, and severe membrane damage. The comprehensive evaluation of salt tolerance ranked the cultivars as follows: ‘Mahaleb CDR-1’ > ‘Daqingye’ > ‘Krymsk5’ > ‘Gisela 6’ > ‘Colt’. This order aligns with the trends observed in plant growth, photosynthetic parameters, and physiological-biochemical indices. As salt concentration increased, the growth rates of plant height and stem diameter, as well as photosynthetic performance, were inhibited in all rootstocks. ‘Mahaleb CDR-1’ was the least affected, followed by ‘Daqingye’ and ‘Krymsk5’, while ‘Gisela 6’ and ‘Colt’ demonstrated relatively weaker salt tolerance. The salt tolerance capacity of the rootstocks was closely associated with the coordinated regulation of osmotic adjustment and antioxidant damage mitigation. These findings provide key physiological insights for the breeding of salt-tolerant rootstock cultivars.

## Figures and Tables

**Table 1:** Comparison of Salt Damage Indices Among Different Cherry Rootstocks under 300 mmol/L NaCl stress. The data are presented as treatment mean ± SD. In the bar chart, different lowercase letters (a, b, c) indicate significant differences at the 0.05 level.

Rootstocks	Salt Damage Index (%)	Ranking of Salt Damage Indices
‘Mahaleb CDR-1’	78.33	1
‘Daqingye’	83.33	2
‘G6’	86.67	3
‘K5’	90.00	4
‘Colt’	98.33	5

**Table 2:** Effects of NaCl Stress on the Biomass of Different Cherry Rootstocks. The data are presented as treatment mean ± SD. Different English letters in the same column indicate a significant difference at the level of 0.05.

Concentration of Stress Agent (mmol·L^−1^)	‘Mahaleb CDR-1’	‘Daqingye’	‘Krymsk5’	‘Gisela 6’	‘Colt’
0 (CK)	1.47 ± 0.0351 cA	1.46 ± 0.05 aA	0.66 ± 0.01 dC	1.00 ± 0.0115 cB	0.57 ± 0.01 dD
50	1.87 ± 0.0252 bA	1.49 ± 0.0551 aB	0.68 ± 0.0058 dD	1.06 ± 0.0115 bC	0.63 ± 0.0115 cE
100	1.92 ± 0.0551 bA	1.51 ± 0.0751 aB	0.72 ± 0.0153 cD	1.28 ± 0.0173 aC	0.64 ± 0.0153 cE
150	2.11 ± 0.0503 aA	1.17 ± 0.0764 bC	1.40 ± 0.0451 aB	1.00 ± 0.0115 cD	0.81 ± 0.0208 aE
200	2.06 ± 0.0416 aA	1.08 ± 0.0058 bC	1.23 ± 0.0289 bB	0.63 ± 0.0252 dE	0.71 ± 0.0252 bD
250	1.90 ± 0.0503 bA	1.02 ± 0.0451 bcB	0.53 ± 0.0058 eD	0.59 ± 0.0351 deD	0.69 ± 0.02 bC
300	1.85 ± 0.0361 bA	0.91 ± 0.18 cC	0.51 ± 0.0058 eD	0.57 ± 0.0252 eC	0.63 ± 0.02 cC

Note: CK, Control. Different lowercase letters indicate significant differences among treatments (*p* < 0.05), while different uppercase letters indicate significant differences among cultivars (*p* < 0.05). The same notation is used in the following tables and figures.

**Table 3:** Membership Function Values and Rankings of Various Physiological and Biochemical Indices under Different NaCl Treatments. The data are presented as treatment mean ± SD. Different English letters in the same column indicate a significant difference at the level of 0.05.

Cultivar	Plant Height	Stem Diameter	Soluble Sugar Content	Soluble Protein Content	MDA Content	SOD Content	Pro Content	CAT Content	POD Content	Biomass	Comprehensive Evaluation	Ranking
‘Daqingye’	0.51	0.20	0.44	0.19	0.63	0.12	0.13	0.11	0.37	0.21	0.29	2
‘Colt’	0.26	0.08	0.51	0.13	0.48	0.12	0.12	0.10	0.36	0.33	0.25	5
‘Krymsk5’	0.32	0.11	0.50	0.16	0.49	0.12	0.12	0.09	0.36	0.32	0.26	3
‘Gisela 6’	0.40	0.17	0.47	0.16	0.55	0.12	0.13	0.11	0.37	0.11	0.26	4
‘Mahaleb CDR-1’	0.58	0.24	0.51	0.19	0.62	0.12	0.14	0.11	0.37	0.47	0.34	1

## Data Availability

The original contributions presented in this study are included in the article/Supplementary Material. Further inquiries can be directed to the corresponding author.

## Supplementary Materials

The following supporting information can be downloaded at: https://www.mdpi.com/article/10.3390/plants15050737/s1, Figure S1. Effect of NaCl stress on soluble sugar content in the leaves of different cherry rootstocks. Figure S2. Effect of NaCl stress on soluble protein content in the leaves of different cherry rootstocks. Figure S3. Effect of NaCl stress on Pro content in the leaves of different cherry rootstocks. Figure S4. Effect of NaCl stress on MDA content in the leaves of different cherry rootstocks. Figure S5. Effect of NaCl stress on catalase (CAT) activity in the leaves of different cherry rootstocks. Figure S6. Effect of NaCl stress on SOD activity in the leaves of different cherry rootstocks. Figure S7. Effect of NaCl Stress on POD activity in the leaves of different cherry rootstocks. Tables S1. Effects of NaCl Stress on Net Photosynthetic Rate (*Pn*) of Different Cherry Rootstocks. Tables S2. Effects of NaCl Stress on Transpiration Rate (*Tr*) of Different Cherry Rootstocks. Tables S3. Effects of NaCl Stress on Stomatal Conductance (*Gs*) of Different Cherry Rootstocks. Tables S4. Effects of NaCl Stress on Intercellular CO_2_ Concentration (*Ci*) of Different Cherry Rootstocks.

## Abbreviations

The following abbreviations are used in this manuscript:

CAT	Catalase
SOD	Superoxide dismutase
POD	Peroxidase
PRO	Proline
MDA	Malondialdehyde
