# Supplementary material for: Integrative Evaluation of Salt Tolerance in Cherry Rootstocks Using Phenotypic and Biochemical Markers
Source: Plants (Basel). 2026 Feb 28;15(5):737. doi: 10.3390/plants15050737 (PMC12987332; doi:10.3390/plants15050737)
Supplement: Supplementary file 1 [file plants-15-00737-s001.zip › plants-4089354-supplementary.pdf]

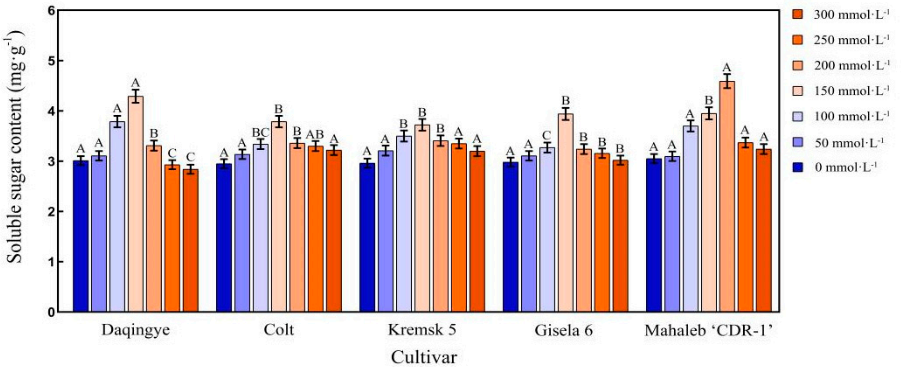

Supplementary Figure S1. Effect of NaCl stress on soluble sugar content in the leaves of different cherry rootstocks ['Daqingye,' 'Colt,' 'Mahaleb CDR-1,' 'Kremsk 5', and 'Gisela 6'].Error bars indicate the SD of treatment means. Different letters on the bars indicate significant differences among the cultivars ($p < 0.05$).

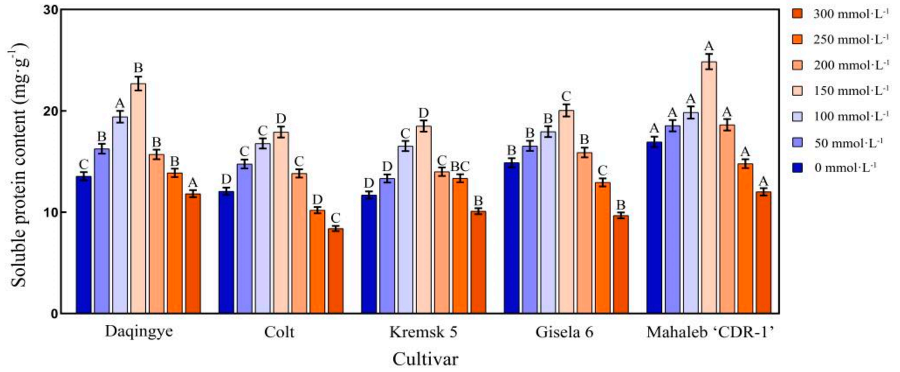

Supplementary Figure S2. Effect of NaCl stress on soluble protein content in the leaves of different cherry rootstocks ['Daqingye,' 'Colt,' 'Mahaleb CDR-1,' 'Kremsk 5' , and 'Gisela 6'].Error bars indicate the SD of treatment means. Different letters on the bars indicate significant differences among the cultivars ($p < 0.05$).

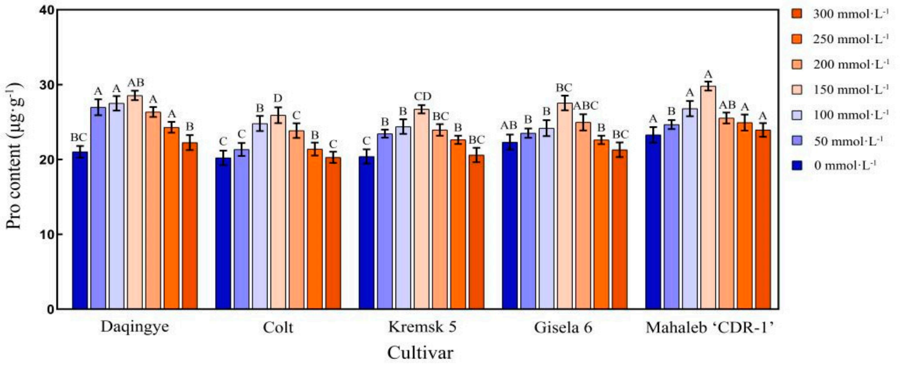

Supplementary Figure S3. Effect of NaCl stress on Pro content in the leaves of different cherry rootstocks ['Daqingye,' 'Colt,' 'Mahaleb CDR-1,' 'Kremsk 5' , and 'Gisela 6'].Error bars indicate the SD of treatment means. Different letters on the bars indicate significant differences among the cultivars ($p < 0.05$).

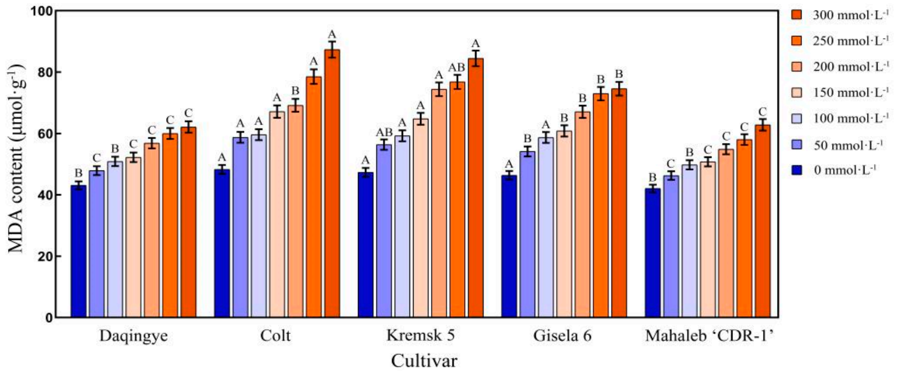

Supplementary Figure S4. Effect of NaCl stress on MDA content in the leaves of different cherry rootstocks ['Daqingye,' 'Colt,' 'Mahaleb CDR-1,' 'Kremsk 5', and 'Gisela 6'].Error bars indicate the SD of treatment means. Different letters on the bars indicate significant differences among the cultivars ($p < 0.05$).

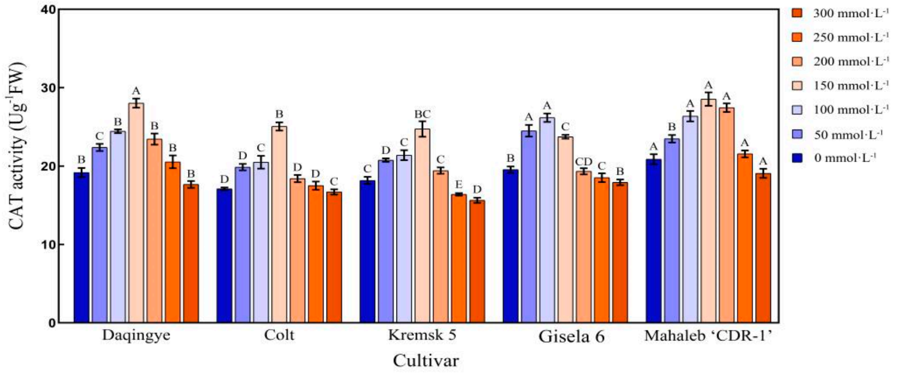

Supplementary Figure S5. Effect of NaCl stress on catalase (CAT) activity in the leaves of different cherry rootstocks ['Daqingye,' 'Colt,' 'Mahaleb CDR-1,' 'Kremsk 5', and 'Gisela 6'].Error bars indicate the SD of treatment means. Different letters on the bars indicate significant differences among the cultivars ($p < 0.05$).

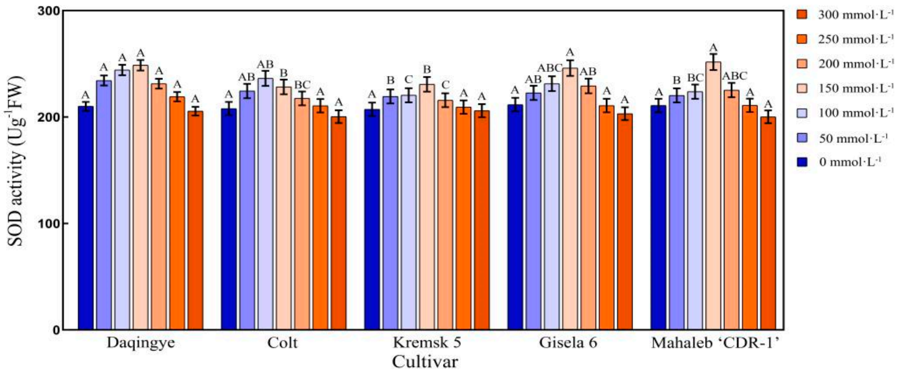

Supplementary Figure S6. Effect of NaCl stress on SOD activity in the leaves of different cherry rootstocks ['Daqingye,' 'Colt,' 'Mahaleb CDR-1,' 'Kremsk 5', and 'Gisela 6'].Error bars indicate the SD of treatment means. Different letters on the bars indicate significant differences among the cultivars ($p < 0.05$).

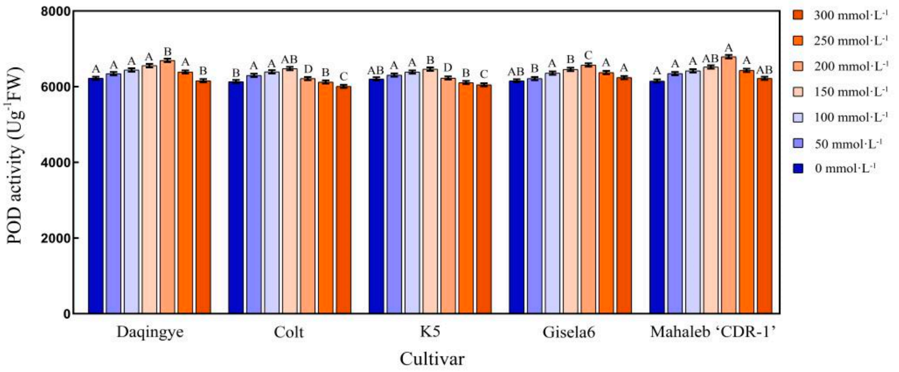

Supplementary Figure S7. Effect of NaCl Stress on POD activity in the leaves of different cherry rootstocks ['Daqingye,' 'Colt,' 'Mahaleb CDR-1,' 'Kremsk 5', and 'Gisela 6'].Error bars indicate the SD of treatment means. Different letters on the bars indicate significant differences among the cultivars ($p < 0.05$).

Supplementary Tables S1. Effects of NaCl Stress on Net Photosynthetic Rate (*Pn*) of Different Cherry Rootstocks.The data are presented as treatment mean ± SD. Different English letters in the same column indicate a significant difference at the level of 0.05.

Concentration of Stress Agent (mmol/L)	'Mahaleb CDR-1'	'Daqingye'	'K5'	'G6'	'Colt'
0 (CK)	9.15±0.326aA	5.483±0.1956aD	9.647±0.341aA	7.363±0.261 aB	6.34±0.221aC
50	8.01±0.281bA	4.577±0.160bD	6.677±0.236bB	7.067±0.246 aB	5.203±0.186bC
100	6.28±0.221cA	4.077±0.141cC	4.337±0.150cC	6.2±0.221bA	4.743±0.166cB
150	5.183±0.186dA	3.707±0.131dD	4.057±0.141 cC	4.863±0.176 cB	4.077±0.141dC
200	4.973±0.176dA	3.677±0.131dC	3.52±0.125 dC	4.007±0.141dB	3.967±0.141dB
250	4.317±0.150eA	3.407±0.121eC	2.72±0.095eD	3.757±0.131 dB	3.647±0.131eB
300	4.047±0.141eA	2.48±0.085fC	2.063±0.070fD	1.713±0.060eE	2.8±0.095fB

Supplementary Tables S2. Effects of NaCl Stress on Transpiration Rate (*Tr*) of Different Cherry Rootstocks.The data are presented as treatment mean ± SD. Different English letters in the same column indicate a significant difference at the level of 0.05.

Concentration of Stress Agent (mmol/L)	'Mahaleb CDR-1'	'Daqingye'	'K5'	'G6'	'Colt'
0 (CK)	3.36±0.150aB	3.81±0.170aA	3.95±0.180aA	3.87±0.170 aA	2.94±0.063aC
50	3.31±0.150 aB	2.61±0.115bC	3.60±0.160bA	3.40±0.155bAB	2.06±0.042bD
100	3.16±0.145abA	2.57±0.115bB	3.39±0.155 bA	3.15±0.145cA	1.90±0.0416 cC
150	2.97±0.135bcA	2.57±0.115bB	3.03±0.135 cA	2.82±0.125 dA	1.69±0.036dC
200	2.83±0.125cdA	2.34±0.105cB	2.39±0.110dB	2.66±0.120dA	1.68±0.036dC
250	2.73±0.125cdA	2.33±0.105cB	2.30±0.100dB	2.37±0.105eB	1.67±0.036dC
300	2.70±0.120dA	2.10±0.090dB	1.73±0.080eCD	1.91±0.090fBC	1.63±0.036dD

Supplementary Tables S3. Effects of NaCl Stress on Stomatal Conductance (*Gs*) of Different Cherry Rootstocks (Unit: $mol{\cdot}m^{-2}{\cdot}s^{-1}$).The data are presented as treatment mean ± SD. Different English letters in the same column indicate a significant difference at the level of 0.05.

Concentration of Stress Agent (mmol/L)	'Mahaleb CDR-1'	'Daqingye'	'K5'	'G6'	'Colt'
0 (CK)	0.16±0.01aB	0.13±0.01aC	0.16±0.01 aB	0.19±0.015aA	0.14±0.01aBC

50	0.15±0.006 bA	0.11±0.01bB	0.14±0.006aA	0.11±0.01bB	0.06±0.006bC
100	0.13±0.006cA	0.10±0.01bB	0.11±0.006 bB	0.10±0.01bB	0.06±0.012bcC
150	0.11±0.006dA	0.08±0.006cB	0.07±0.006 cB	0.08±0.006cB	0.05±0.006bcdC
200	0.08±0.006eA	0.07±0.006cAB	0.06±0.017cdBC	0.07±0.006cdAB	0.04±0.006cdeC
250	0.07±0.006efA	0.06±0.006 cAB	0.05±0.006dB	0.06±0.006dAB	0.04±0.01 deC
300	0.07±0.006fA	0.05±0.012dAB	0.04±0.012dB	0.04±0.006eB	0.04±0.006eB

Supplementary Tables S4. Effects of NaCl Stress on Intercellular CO_2 Concentration (*Ci*) of Different Cherry Rootstocks (Unit: $\mu mol \cdot m^{-2} \cdot s^{-1}$).The data are presented as treatment mean ± SD. Different English letters in the same column indicate a significant difference at the level of 0.05.

Concentration of Stress Agent (mmol/L)	'Mahaleb CDR-1'	'Daqingye'	'K5'	'G6'	'Colt'
0 (CK)	312.66±7.84cB	327.42±8.21dA	304.74±7.64eB	278.49±6.99cC	260.13±6.53fD
50	293.19±7.35 dAB	303.88±7.63eA	282.32±7.08fB	257.45±6.46dC	224.19±5.62gD
100	287.64±7.21dB	290.91±7.30ebB	312.78±7.84 eA	234.52±5.88eC	288.23±7.23eB
150	267.31±6.70eC	330.99±8.30dB	350.64±8.79 dA	265.12±6.65dC	323.28±8.11dB
200	293.97±7.37dC	361.48±9.07cB	377.17±9.46cA	289.21±7.25cC	351.70±8.82cB
250	342.27±8.58bC	377.14±9.46bB	394.25±9.89bA	303.50±7.61bD	394.86±9.91bA
300	362.52±9.09aD	401.59±10.07aC	420.38±10.54aB	338.79±8.50aE	445.01±11.16aA
